# Transmission and Control of SARS-CoV-2 in the Food Production Sector: A Rapid Narrative Review of the Literature

**DOI:** 10.3390/ijerph191912104

**Published:** 2022-09-24

**Authors:** Paniz Hosseini, William Mueller, Sarah Rhodes, Lucy Pembrey, Martie van Tongeren, Neil Pearce, Miranda Loh, Tony Fletcher

**Affiliations:** 1 Department of Public Health, Environments and Society, London School of Hygiene and Tropical Medicine, London WC1E 7HT, UK; 2 Institute of Occupational Medicine, Edinburgh EH14 4AP, UK; 3 Division of Population Health, Health Services Research & Primary Care, University of Manchester, Manchester M13 9PL, UK; 4 Department of Medical Statistics, London School of Hygiene and Tropical Medicine, London WC1E 7HT, UK

**Keywords:** COVID-19, COVID transmission, food production sector, occupational health, food and drink production

## Abstract

This review aimed to provide an overview of the literature assessing the extent of COVID-19 transmission in the food processing sector along with the risk factors associated with COVID-19 infection/mortality rates in this setting, and the preventive measures used to reduce transmission. An electronic search was conducted using scientific databases, including Web of Science, OVID, PubMed and MedRxiv. The search strategy identified 26 papers that met the inclusion criteria. Six of these studies were based in the UK and the country with the most papers was the USA, with a total of nine papers. Findings showed some evidence of a high transmission level of SARS-CoV-2 within some areas of the food production sector. Risk factors associated with the spread included ethnicity, poor ventilation, lack of social distancing and lack of sick pay. The preventative measures included/recommended were social distancing, testing, adequate ventilation, cleaning regimes and access to PPE. Additional research focusing on the food production sector could show the potential variations in transmission and risk between each sub-sector. Future research focusing on the application of various preventative measures and their efficacy by sub-sector would be beneficial, while further qualitative research could help provide in-depth information regarding knowledge gaps.

## 1. Introduction

Coronavirus disease 2019 (COVID-19) is an infectious disease caused by the severe acute respiratory syndrome coronavirus 2 (SARS-CoV-2) [1]. It is a novel RNA coronavirus from the same family as SARS-CoV and Middle East Respiratory Syndrome coronavirus (MERS-CoV) [2]. Since the beginning of the COVID-19 pandemic, research has been conducted to understand more about the virus’s transmission routes. The risk of transmission is increased when standing close to a person who is infected [3]. It is also now accepted that the primary transmission route appears to be through close contact human-to-human aerosol transmission, which can occur through contaminated droplets, hands or surfaces [2]. The virus has also been found to last several hours on different surfaces, however, it is uncertain how much surface to eyes, nose or mouth transmission is likely to contribute to outbreaks [3]. Importantly far-field airborne transmission can also occur through virus-laden aerosols emitted from an infected person, which is a particular problem in indoor or enclosed environments with crowding or poor ventilation [4].

It is clear that the COVID-19 pandemic has been having a negative effect in many workplaces, where employers have struggled to effectively exercise their legal duty to protect staff from harm in the workplace [5]. Millions of workers have jobs that cannot be conducted at home and in the UK alone, 33% of the total workforce were identified as key workers during the pandemic [6]. A number of publications have highlighted reports of hotspots and evidence of COVID-19 risks being raised in the food production sector [6]. This review aims to provide an overview of the existing literature to assess the evidence on COVID-19 cases, infection and mortality rates, potential transmission risk factors, and preventative measures within the different areas of the food production sector. The research questions that we aim to address in this review include:

1. What is the evidence for an increased risk of infection, outbreaks and COVID-19 mortality rates in the food production sector compared to other sectors?
2. Which risk factors contribute to any elevated COVID-19 infection and mortality rates in the food production sector?
3. Which preventative measures/risk mitigation strategies have been taken to reduce COVID-19 in the food production sector, and which have shown to be effective?

## 2. Materials and Methods

An electronic search was conducted using scientific databases, including Web of Science, OVID, PubMed, Scopus, IEEE Xplore and MedRxiv (last search 28 October 2021) to gather the existing literature on this topic. The search terms included can be found in the appendix. Other relevant studies identified outside of this search were also included in our review. In the case of finding the papers, the term “food production sector” also included the production of drinks and beverages.

The inclusion criteria consisted of: (1) papers involving the level of transmission in the food production sector (including the risk of infection, outbreaks/cases and mortality rates), (2) papers with information on factors that are linked to an increased risk of COVID-19 infection in this sector, and (3) studies focusing on preventative measures or risk mitigation strategies in the sector. As there were a limited number of food-sector-specific studies, papers that involved a broad range of work sectors (as well as the food production sector) were also included, as were studies based outside of the UK.

## 3. Results

### 3.1. General Findings

Our search retrieved 26 papers that fit the inclusion criteria for this review (Table 1). The country with most publications was the USA (*n* = 9) [7,8,9,10,11,12,13,14,15], while others were based in the UK (*n* = 6) [16,17,18,19,20,21], France (*n* = 1) [22], Ireland (*n* = 1) [23], Spain (*n* = 1) [24], Germany (*n* = 1) [25], Greece (*n* = 1) [26], Sweden (*n* = 1) [27] or focused on a more global perspective (*n* = 4) [28,29,30,31]. One study focused on both the USA and the UK [32].

Table 1 presents the details of the papers extracted for this review.

### 3.2. What Is the Evidence for an Increased Risk of Infection, Outbreaks and COVID-19 Mortality Rates in the Food Production Sector Compared to Other Sectors?

Evidence on outbreaks and infection and mortality rates within the food production sector in the UK has been limited. Many of the papers which focused on the transmission levels within the food production sector were US-based studies (*n*= 7) [7,8,9,10,11,13,15]. Of these, the Centres for Disease Control and Prevention (CDC) published several papers covering the number of cases in various factories across the USA.

#### 3.2.1. Meat and Poultry Facilities

One of these CDC papers included COVID-19 among workers in meat and poultry processing facilities across 19 states in April 2020 [9]. The study showed that across the 19 states, there were a total of 4913 (3.0%) confirmed COVID-19 cases and 20 (0.4%) COVID-19 related deaths. 

Another study published by the CDC also analysed transmission among meat processing workers in Nebraska, along with the effectiveness of risk mitigation measures [10]. The study found that out of the 26,000 workers in meat processing factories across the state, 5002 (19%) were diagnosed with COVID-19 from March to July 2020. They also found that the attack rate during this time period was more than double the 9.1% attack rate reported in a multistate analysis of meat processing facilities across the USA.

House et al. [11] performed a retrospective cohort study of patients less than 65 years of age attending an emergency department (ED) with COVID-19 symptoms in the USA between March–May 2020. They found that amongst all the patients, 8.4% stated they were potentially exposed by working in a meatpacking plant. Out of the 582 patients in the ED, 74% of meatpacking plant exposed patients tested positive for COVID-19, while 12% of those without a meatpacking plant exposure tested positive. However, this large difference between the two groups could possibly be explained by the overall small sample of individuals from the former group compared to the latter. This can also be seen in the multivariable model produced in the study, which found that despite having higher COVID-19 positivity rates, meatpacking plant exposed individuals had similar rates of hospital admissions to individuals who were not exposed. They also concluded that in-hospital mortality did not vary significantly by meatpacking plant exposure. Ethnicity seemed to play a significant role, with figures showing that 57.1% of individuals with a meatpacking exposure were Hispanic/Latino, but only made up 11.8% of the non-meat packing exposure group [11].

Steinberg et al. [13] also investigated an outbreak among employees at a meat processing facility in South Dakota between March and April 2020. They found that of the 3635 people working in Facility A (a facility with 38 departments that harvested and processed animals during two shifts per day), there were a total of 929 (25.9%) COVID-19 cases and that out of the 2199 COVID-19 cases identified among community residents, facility A employees represented 920 (41.8%) of them. The highest attack rates also occurred in the Cut (30.2%) and Harvest (29.4%) department groups, while the attack rate remained higher for nonsalaried employees (26.8%). However, this difference between nonsalaried and salaried employees could be associated with salaried employees having access to workstations that could be adjusted to maintain social distancing, something that nonsalaried employees were not given access to [13].

Outside of the USA, Mallet et al. [22] investigated a COVID-19 cluster that occurred in the pork section of a plant in France during May 2020. In total, there were 140 occupational cases identified during this period, of which 27 were identified through hospital or outpatient sampling. Although there were no mortality rates, four individuals were hospitalised (2.9%), two of whom were admitted to the intensive care unit. All four cases that were hospitalised worked in the deboning and cutting department of the plant. Foreign-born workers accounted for half of the total cases (52.1%) compared to a quarter (25.4%) of non-cases and were more likely to be placed in the deboning and cutting department. The majority of cases were employed by subcontracting companies (50.7%) or were temporary workers (30.7%). The attack rate in the study population was also 11.9% but was 16.6% among workers of the deboning and cutting department. It was concluded that there was a significantly increased risk of SARS-CoV-2 infection for workers of subcontractors and some foreign-born workers [22].

A paper published by the national COVID-19 outbreak monitoring group [24] reported outbreaks notified at the national level in Spain during the summer of 2020, while also describing settings where outbreaks were most frequently identified [24]. They found that out of the outbreaks which were linked to occupational settings (representing 20% of all active outbreaks), the ones related to workers in the agricultural/fruit and vegetable sector were the most frequent, with 31 active outbreaks and around 500 active cases (“active” defined as new cases diagnosed within the last 14 days). In total, this sector had 45 outbreaks and 1022 cases during this period. Workers at slaughterhouses/meat processing plants were the second most affected group, with 12 active outbreaks and 360 active cases identified. This group also had a total of 19 outbreaks and with a total of 767 cases within this period. This was substantially higher than other occupational settings, such as long-term care facilities and healthcare facilities, which made up 7% and 3% of the active outbreaks, respectively [24].

VanderWaal et al. [14] modelled transmission dynamics and effectiveness of worker screening programs for COVID-19 in three different pork-processing plants during spring of 2020 in the USA. One of the plants (“plant B”) was located in a region with high levels of community transmission early in the pandemic, which they believe was a factor for the steep epidemic curve within the plant during late April. Plant C was the only plant to offer company-sponsored PCR-testing for individuals who had mild signs of the virus, and stated that this caused further documenting of cases and reporting of an apparent larger outbreak, with a cumulative of ~25% of workers clinically affected. They also found that plant C had a policy that asked all household and carpool contacts of potential cases to self-isolate at the same time as the employee showing clinical symptoms. VandelWaal et al. [14] concluded that it was difficult to determine whether Plant C experienced a larger outbreak than Plant B, or if they simply had better documentation of cases from the PCR testing available for symptomatic individuals [14].

Walshe et al. [23] described a retrospective outbreak investigation in a meat processing plant in Ireland, along with a description of the measures taken to prevent future outbreaks. They found that across a five-week period, the plant had a total of 111 confirmed positive asymptomatic cases and an estimated attack rate of 38%. Mass screening was provided four weeks after the outbreak, where they found a further thirty-two positive cases, of which 50% consisted of workers who were based in the boning hall of the plant. After carrying out various risk assessments and air quality monitoring in the boning hall, Walshe et al. found that this area of the plant showed a gradual build-up of carbon dioxide and aerosol particles over the course of a work shift. They confirmed that this area was poorly ventilated and was highly favourable for aerosol transmission of COVID-19 [23]. However, the high number of cases from the boning hall could also be explained by the fact that this area had the greatest number of workers when compared to other production areas [23]. 

Conversely, a study focusing on COVID-19 mortality across occupations in Sweden found that there were 0 deaths reported in the meatpacking sector, making it the only occupation in the database with 0 deaths [27]. In contrast, the authors found mortality rates to be highest among taxi and bus drivers, who had over four times the risk than that of skilled workers in IT, economics or administration jobs. However, the analysis was limited to workers who lived in the same household with an elderly person in the household, making it a very selected group [27]. Similarly, when adjusting for socioeconomic factors, the authors concluded that there were no occupational groups with clearly elevated COVID-19 mortality [27].

#### 3.2.2. Other Food Production Facilities

Many papers focused on food processing relative to other sectors or focused on comparing risks across different occupational sectors. Of these, Waltenburg et al. [15] outlined COVID-19 cases among workers in various food processing, food manufacturing and agriculture workplaces in the USA. They found that from March to May 2020, there were a total of 742 food manufacturing and agriculture workplaces affected, with a total of 8978 confirmed COVID-19 cases among workers and 55 (0.6%) related deaths across the USA [15].

A report by Bui et al. [7] analysing racial and ethnic disparities among COVID-19 cases in Utah from March–June 2020 found that the manufacturing sector, along with wholesale trade, had some of the highest workplace outbreak-associated cases when compared to other sectors. For example, the manufacturing sector had a total of 43 (20%) workplace outbreaks and 467 (20%) workplace-associated cases, while the wholesale trade industry had a total of 29 (14%) outbreaks, of which 200 (14%) were workplace-associated cases. This was significantly higher than other occupational settings, including health care and assistance, which had a total of 5 outbreaks (2%) and 21 (2%) workplace-associated cases [7]. A study by Chen et al. [8] investigating COVID-19 mortality among Californians also found that workers in the food and agriculture sector had a 31% increase in relative excess mortality between June and July 2020. Excess deaths within this sector were also significantly higher than in sectors such as government and community, health/emergency and retail. Other than the food and agriculture sector, rates were also high in the transportation/logistics sector (31%, 91 per 100,00); manufacturing (24%, 61 per 100,000) and facilities (23%, 83 per 100,00). Chen et al. concluded that the pandemic’s effects on mortality have been greatest among essential workers, particularly for those in the food/agriculture sectors, and specifically for Latino and black workers in this sector, who had a 59% increase in mortality when compared to other ethnic groups [8].

In the UK, the Office for National Statistics (ONS) published COVID-19 related mortality rates within different occupations between March and December 2020 [21]. While figures for the food production sector specifically had not been highlighted, they found that within the process, plant and machine operatives, there were a total of 827 deaths for men (52.8 deaths per 100,000 males), making it the third-highest mortality rate out of the nine major occupational groups for men (elementary occupations having the highest mortality rate, followed by the caring, leisure and other service occupations). For women, this group had the highest rates of COVID-19 related deaths when compared to the nine other major occupational groups, with a total of 57 deaths (33.8 per 100,000). They also found that there were 103.7 deaths per 100,000 males in the food, drink and tobacco process operatives during this same period [21].

A study by Chen et al. [16] analysed Public Health England (PHE) HPZone data on COVID-19 outbreaks in workplaces across 9 different regions in England. They calculated outbreak rates and infection attack rates associated with different occupational groups, one of which included manufacturers and packers of food. Of the 6998 workplaces for manufacturers and packers of food in England, there were a total of 117 outbreaks during this time period, resulting in an outbreak rate of 1672/100,000. This was higher than any other industrial sector and was consistent over seven of the nine geographical locations. While the attack rate varied, and typically increased as the size of the enterprise decreased, they concluded that it was higher amongst workers in close contact services, restaurants and manufacturers and packers of non-food products [16].

Kotsiou et al. [26] analysed COVID-19 prevalence in Greece pre-lockdown and during lockdown across various occupations using repeated Antigen-Based Rapid Diagnostic Testing. They found that employees working in the catering/food sector (term not defined but often referred to as the “food processing sector”) experienced some of the highest odds of COVID-19 positivity than those employed in other jobs. Their data showed that 35% of the 48 individuals working in the food sector tested positive for COVID-19, making it the highest figure out of all the occupations. However, the sample size of this group was small, with only a total of 48 workers in the food sector being included in the study, making up only 5% of the total sample size [26].

Some studies found that other occupational groups were at a higher risk of COVID-19 infection and mortality. For example, a study by Mutambudzi et al. [19] analysing the occupational risk of severe COVID-19 in a study of 120,075 UK Biobank participants found that 271 had severe COVID-19 symptoms. Of these, healthcare workers, social workers and education workers were identified as the highest risk group, but only 0.2% of food workers were found to have severe COVID-19, along with 0.4% of those in the process, plant and machine operatives [19]. However, when using the Standard Occupation Classification (SOC) major occupational groups list, they found that process, plant and machine operatives were considered high risk, particularly when compared to managers and senior officials. Nonetheless, they stated this was mostly explained by socio-economic factors [19].

Similarly, a study by Hiironen et al. [17] used three retrospective studies (late August, September and October 2020) and case data from the NHS Test and Trace programme to analyse transmission and occupational exposures associated with COVID-19 cases. They found that across all study periods, there was strong evidence showing that those working in healthcare, social care, hospitality and warehouse settings had increased odds of being a COVID-19 case. There was limited evidence of any elevated risk for food production and agriculture workers (OR of 1.20, 1.84 and 0.90 in the three different time periods listed above), however, the risk for this group was still considerably higher than occupations such as education, retail, work-related travel and arts, entertainment and recreation. Nonetheless, the study has its limitations due to potential selection bias as 85% of the cases transferred to the NHS Test and Trace app were reached by the contact tracing programme, and those who do not engage in contact tracing may differ from others in terms of their exposure. Similarly, the Test and Trace app would not pick up those not using the app, and would not identify asymptomatic cases, which may be more prevalent in certain occupations [17]. A paper by Nafilyan et al. [20] also analysed the hazard ratios for COVID-19 related deaths for adults aged 40–64 years in England. They found no evidence of elevated risks for males (HR 1.15 [0.89–1.50]) or females (HR 1.15 [0.750–1.77] working in food production compared to non-essential workers after full adjustment for potential confounders. Instead, they noted that the highest risk occupations appeared to be individuals working for taxi/cab/chauffeur services, followed by other elementary occupations, care workers and home carers [20].

Table 2 provides a breakdown of the total number of COVID-19 related cases and deaths reported by each study. 

### 3.3. Which Risk Factors Contribute to Any Elevated COVID-19 Infection and Mortality Rates in the Food Production Sector?

Several papers found factors that they reported could increase the risk of COVID-19 spreading in the workplace and amongst workers in the food production sector. Table 3 summarises the main findings related to the risk factors found in the studies. 

#### 3.3.1. Transport

Transport was identified as a risk factor in 4 of the papers [12,22,28,32]. The main finding related to transport was that many individuals working in the food processing sector were likely to travel to and from work with people from different households, hence increasing the risk of transmission. However, this factor was not analysed in much depth in these papers, suggesting that the evidence behind this may be lacking. For example, Anand et al. [32] concluded that there was “some, often weaker, evidence that income, car ownership, used of a shared kitchen, university degree type (…) are predictors of COVID-19 transmission”, suggesting more evidence is required when referring to transport as a risk factor for transmission [32].

#### 3.3.2. Shared Accommodation

This was only identified as a risk factor in one of the papers [22] and was found to be more common for foreign-born workers.

#### 3.3.3. Temporary Workers

Mallet et al. [22] found that the majority of COVID-19 cases found were amongst employees that were employed by subcontracting companies or were temporary workers. Steinberg et al. [13] also found that cases were highest amongst nonsalaried employees, as salaried employees were more likely to work in low-risk areas of the site [13].

#### 3.3.4. Income and Sick Pay

##### Sick Pay

The impact of income/lack of sick pay on the risk of COVID-19 was not analysed in many papers. However, Aday and Aday [28], Moore et al. [18] and Anand et al. [32] found some significant links. For example, there was some evidence suggesting that the majority of workers in the food manufacturing sector have a lower income and do not have health insurance/paid sick leave, a factor that suggests workers are more likely to go to work even when they are feeling unwell/experiencing COVID-19 symptoms [28]. Anand et al. [32] also found that workers in the lowest household income groups were at higher risk of COVID-19 infection within both the USA and UK, though they noted that this evidence was “weak” [32]. Alternatively, Moore et al.’s [18] findings showed that changes were made for workers’ sick pay entitlements in the UK food manufacturing sector during the course of the pandemic, (see Table 3) [18]. Bui et al. also found that there may be a significant link between lack of sick pay and ethnicity, noting that there was a lack of job flexibility for Hispanic and non-white workers in their study. They suggested that this lack of job flexibility, particularly around unpaid or punitive sick leave policies, may prevent workers from staying home when unwell. In turn, this could result in further workplace exposures and more severe COVID-19 outcomes [7].

#### 3.3.5. Environmental Factors

Environmental factors, such as poor ventilation, lack of social distancing and cold and humid environments inside food-processing facilities, may be associated with increased transmission of COVID-19, as findings referenced by Aday and Aday [28], which referred to Chin et al. [33]’s study on COVID-19 stability in different environmental conditions. Chin et al. [33] found that SARS-CoV-2 is highly stable at 4 °C, but sensitive to heat, suggesting that workers placed in areas with such temperature levels may be at higher risk of COVID-19 transmission [33].

Günther et al. [25] also found in their outbreak investigation of a German meat processing plant that certain environmental conditions mixed with a lack of social distancing between workers, created further aerosol transmission. They also found that the transmission of the virus could occur over distances of at least 8 metres in conditions with low air exchange and high rates of recirculated unfiltered air [25]. They suggested that a physical distance of 2 m would not be enough to prevent transmission, especially in environmental conditions with poor ventilation in place, but would instead require additional measures such as improved ventilation and airflow, installation of filtering devices or use of high-quality face masks [25]. Similarly, Walshe et al. found that in the meat processing plant they studied, there was no extraction of air from the boning hall and that air exchange was limited. They also found decay of CO_2_ concentration during the two break periods, which explained the gradual rise in particle counts and CO_2_ over each working shift. They stated that this was indicative of poor ventilation [23].

Other studies, such as those by Steinberg et al. [13] and Mallet et al. [22] also found that groups of people who were placed in certain areas of the workplace were more likely to test positive for COVID-19. In particular, they found that cases were highest in areas such as the deboning and cutting departments, as they were also areas where social distancing was less likely to be maintained [13,22]. Mallet et al. also found that there was an association between Eastern European staff working in the cutting department testing positive for SARS-CoV-2 and suggested that this may be due to language barriers in place, which may have caused less social distancing and mask wearing [22]. Steinberg et al. also found that contact between employees in common areas, such as cafeterias and locker rooms may have facilitated spread among employees in different departments in the meat processing facility studied. They suggested that maintaining distancing and introducing staggered shifts and break times may reduce transmission amongst employees in these areas [13].

#### 3.3.6. Ethnicity

Out of the 14 studies which analysed the risk factors associated with COVID-19 transmission in the food processing sector, 9 identified ethnicity as a potential contributing factor. All of these studies found that migrant or minority ethnic groups were at substantially higher risk of being infected with COVID-19, and mainly cited that this was due to structural factors within the workplace, such as being placed within work in areas which increased the risk of transmission (e.g., working in cold-temperature areas), or due to contributing factors such as lack of sick pay being offered to these groups [7]. One study [12] also found that ethnicity and transport were both risk factors, with the odds of foreign-born workers commuting to work with individuals from outside their household being around 1.9 times the odds for US-born workers [12]. Similarly, House et al.’s [11] study found that overall, patients in the emergency department from meatpacking plants were more likely to be Black or Hispanic, compared to patients without this occupational exposure [11]. 

However, it is important to note that ethnicity was often found as a risk factor when other factors were taken into account, (such as environmental factors). It is therefore unlikely that COVID-19 risk increased as a result of an individual’s ethnicity directly, but rather increased their chances of working and being placed in environments that caused an unfavourable risk of transmission, such as areas with poor ventilation and/or lack of room for effective social distancing [7,8,12]. As suggested by Bui et al. (2020) [7], the racial and ethnic disparities in workplace associated COVID-19 cases in the food sector could suggest a disproportionate risk for COVID-19. This could potentially be driven by the longstanding health and social inequities resulting in the over-representation of certain ethnic groups in such occupations [7]. Similarly, Rubenstein et al. [12] noted that structural factors were more apparent than behavioural factors amongst foreign-born workers, who were more likely to share transportation and come from larger households. They also found that structural factors within the workplace were more evident for foreign-born workers, who had a higher chance of being placed on the production floor, an area with the highest SARS-CoV-2 attack rates [12]. Chen et al. (a) also suggested that inequalities in risk between different groups may be exacerbated by underlying structural inequities, such as immigration status or poverty [8].

Conversely, it may be difficult to suggest that individuals from different ethnic backgrounds were at higher risk of catching the virus, given that many of the papers focused on sites which also had a higher proportion of migrant employees or employees from different ethnic backgrounds employed from the beginning. However, as suggested by Mallet et al. [22], foreign-born individuals make up a large section of the meat and poultry workforce, therefore it is essential to implement the appropriate COVID-19 risk management measures moving forward [22].

### 3.4. Which Preventative Measures/Risk Mitigation Strategies Have Been Taken to Reduce COVID-19 in the Food Production Sector, and Which Have Shown to Be Effective? 

Several studies found in this literature review focused on preventative measures/risk mitigation strategies in helping to reduce COVID-19 transmission in different areas of the food production sector. Summaries of each of the risk mitigation strategies and their effectiveness can be seen in Table 4. 

According to the Hierarchy of Controls in occupational health, eliminating the source of hazards (in this case COVID-19) and/or substitution of the hazard are some of the most effective ways of eliminating the hazards/risk, while actions and measures which rely on an individuals’ behaviour are often seen as the least reliable way of limiting risk [34]. In this case, it can be implied that risk mitigation strategies such as adequate screening/testing for COVID-19, and providing generous sick leave for individuals who have symptoms, may be some of the most effective preventative measures for individuals in the food processing sector, as they are both measures which can physically remove the hazard. Nevertheless, while factors such as elimination and substitution are the most effective at reducing hazards, they also tend to be the most difficult to implement in an existing process. This was found in papers focusing on the risk mitigation measures mentioned, with Aday and Aday [28] suggesting that regular testing/screening, though effective, can be expensive and time-consuming. Similarly, VanderWaal et al. [14] found that frequent testing may not always prevent a large outbreak within food-processing workforces, given that the number of cases could be related to other factors, including community exposure/outbreaks [14].

Another effective control outlined in the hierarchy is engineering controls. Ventilation could fall under this category and can reduce the risk of far-field transmissions. While increasing effective ventilation in food processing settings could be more expensive, it can be more cost-effective in the long run due to the growing evidence that increasing ventilation can substantially reduce far-field COVID-19 transmission [10]. 

Other risk mitigation strategies listed, such as respiratory protective equipment (RPE) and face coverings, social distancing and adequate hygiene practice, would likely fall at the bottom of the hierarchy as they require individuals to change the way they work and use RPE and face coverings adequately. While studies did outline the importance of providing adequate RPE face coverings to workers and ensuring social distancing is in place, there were some limitations and problems which could still be associated with them. For example, Herstein et al. [10] concluded that while wearing face masks is one of the most effective tools in reducing COVID-19 transmission, the effectiveness of a universal mask policy would only work if workers are being educated and adhering to proper mask use. They also stated that while physical barriers and social distancing may help reduce near-field transmissions, the low temperatures and limited fresh air supply in meat processing factories could facilitate longer-range aerosol transmission, hence increasing the risk of infection amongst workers regardless [10]. Similarly, papers focusing on hygiene practices, such as wiping high touch surfaces and regular cleaning regimes, were mainly drawn from grey literature and online reports that focused on COVID-19 prevention as a whole, rather than focusing on the food production sector. Similarly, the literature referenced did not include any which analysed the efficacy of these preventative measures within different areas of the food production sector. 

It is clear that various strategies must be adopted in preventing infection, rather than the adoption of just one of these risk mitigation methods. Herstein et al. [10] state that challenges in the meat processing facilities cannot be addressed with only one or two measures, but rather require multi-layered interventions that target a range of strategies in reducing the transmission of COVID-19. Nakat and Bou-Mitri [29] also emphasised the importance of hand-washing alongside all other preventative methods, along with further training and effective communication between employers and workers in food facilities/factories [29]. Of all the studies focusing on prevention, only one study actively studied the risk mitigation strategies used at a site to assess the effectiveness of each measure in the setting [23]. By analysing these risk mitigation strategies, they found that the site was able to effectively control the spread of the virus once guidance from public health authorities were adapted and optimised to fit the needs of the site, particularly by plant emergency response teams [23].

## 4. Discussion

This review provides an up-to-date overview of the evidence of SARS-CoV-2 transmission, risk factors and prevention in the food sector. While it has given insightful information, a number of key gaps have been identified: (1) there remains a lack of evidence on the level of COVID-19 transmission and risk of infection within the food sector that is UK specific; (2) a very small number of studies have focused on transmission levels and cases found in the different areas of the food sector, with the majority focusing on various occupations at the same time, particularly shifting their focus onto perceived “higher risk” jobs, such as those in the healthcare and education sectors; (3) most studies analysing the transmission levels within the food sector did not include any personal accounts from staff members and managers and predominately used quantitative methodologies; (4) existing food production sector-specific studies mainly focused on meat/poultry facilities and (5) while there were studies highlighting the specific risk factors and the mitigation measures that can be taken, there remains little evidence on how these measures and factors have been used by different areas of the food production sector. Therefore, it is difficult to establish the efficacy of the preventative measures highlighted.

## 5. Conclusions

In summary, there was some evidence showing high transmission of SARS-CoV-2 within the food sector including the risk factors associated with the spread, and the suggested preventative measures to be taken. While many studies did not only focus on the food processing sector as a whole, the ones that did found that individuals working in this industry were at significantly high risk of COVID-19 infection. In particular, there were high infection rates and outbreaks reported for various meat/poultry sites across the USA. Some risk factors associated with transmission included environmental factors such as cold and humid environments, lack of social distancing and poor ventilation. There was also some evidence suggesting that lack of sick pay played a significant role, and that an individual’s ethnic background often affected structural factors within the workplace, which may have put certain groups of people at higher risk of infection. Various risk mitigation strategies were also outlined for the sector, including social distancing, cleaning and disinfecting high-touch areas, enhancement of ventilation and providing more community and work-based testing. There remains a lack of strong evidence behind the risk associated with sharing accommodation/transport to and from work. Similarly, many of the prevention methods outlined were recommended through general COVID-19 risk assessments provided by various organisations, rather than preventative measures that were analysed within the food production sector specifically. 

## 6. Future Directions

Further research focusing on the application of suggested mitigation measures and their efficacy is needed to understand which methods work well in the sector. In terms of reducing transmission and risk, particular attention must be paid to implementing COVID-19 risk management measures which are tailored to specific sites and their own personal situations. For example, sites experiencing more cases amongst certain ethnic groups should ensure certain groups are not being disproportionately placed in higher-risk work environments. Managers in the food production sector could also ensure better systems are in place to tackle any of the structural problems being faced by ethnic minorities, such as providing language translations to ensure better communication of mitigation measures, ensuring sick pay entitlement is the same as other workers and providing solutions to car-sharing arrangements. Similarly, further work and funding should be prioritized for installing effective ventilation systems across multiple sub-sectors, particularly those that have been unable to implement other measures, such as effective social distancing. Specific attention should be given to placing ventilation and air filtration in areas of the workplace which have found to be at higher-risk of transmission, such as boning halls in meat processing facilities. Given that most workers in this sector are essential workers, it is important to ensure the prevention of transmission is prioritized for such groups for future epidemics and pandemics. In doing so, it seems applicable to ensure testing, vaccinations and access to PPE are also provided as soon as possible, while any structural and/or factors outside of the workplace are taken into account. Given the number of quantitative methodologies used in a majority of the studies, further qualitative research would help in identifying key gaps and providing in-depth information regarding enablers and barriers to transmission, risk factors and mitigation moving forward, Finally, research focusing on extracting varied information on the levels of transmission and risk factors is required across all areas of the food production sector, as this likely varies by each type of facility, sub-sector and geographical area. 

## Figures and Tables

**Table 1:** Characteristics of the papers included in the review.

Study	Peer Reviewed?	Location	Summary and Study Design	Sub-Sector	Area of Focus
Aday & Aday (2020) [28]	yes	Global	Literature review on the effects of COVID-19 on food production, processing, distribution and demand.	Food Processing	Risk factors and prevention
Anand et al. (2020) [32]	Yes	USA and UK	Discussion paper—provides evidence for work and personal predictors of COVID-19 transmission.	Factories	Risk factors
Bui et al. (2020) [7]	Yes	Utah, USA	Multiple sector study—analyses the racial and ethnic differences in COVID-19 cases and occupation.	Manufacturing; meat processing	Transmission and prevention
Billingsley et al. (2021) [27]	Yes	Sweden	Sector-specific study—analyses mortality across occupations.	Meat packing	Transmission and mortality
Chen et al. (2021a) [8]	Yes	California, USA	Sector-specific study. Estimates of excess mortality among Californians 18–65 years of age by occupational sector	Food and agricultural workers	Transmission
Chen et al. (2021b) [16]	Pre-print	UK	Epidemiological surveillance data—Analysed Public Health England (PHE) HPZone data on COVID-19 outbreaks in workplaces between 18 May–12 October 2020.	Manufacturers and packers of food	Transmission
Dyal et al. (2020) [9]	Yes	USA	Sector-specific study. Reports of the number of COVID-19 cases across meat and poultry facilities.	Meat and Poultry processing	Transmission
Gunther et al. (2020) [25]	Yes	Germany	Sector-specific study. Describe a multifactorial investigation of the COVID-19 outbreak in a large meat processing complex in Germany.	Meat processing plants	Transmission
Herstein et al. (2021) [10]	Yes	Nebraska, USA	Sector-specific study—Details demographics and outcomes of severe COVID-19 cases among workers in Nebraska meat processing facilities.	Meat processing	Transmission and prevention
Hiironen et al. (2020) [17]	Pre-print	UK	Retrospective study—analyses occupational exposures which were associated with COVID-19 between Aug–Oct 2020.	Food production workers	Transmission and risk factors
House et al. (2021) [11]	Yes	USA	Retrospective cohort study—characterises the association between meat packing plant exposure and clinical outcomes amongst emergency department patients with COVID-19.	Meatpacking	Transmission and prevention
Kotsiou et al. (2021) [26]	Yes	Greece	Sector-specific study. Investigates the prevalence of COVID-19 changes amongst different occupations during lockdown.	Catering and food sector	Transmission and prevention
Mallet et al. (2021) [22]	Yes	France	Sector-specific study. Analyses risk factors and level of transmission for a COVID-19 cluster detected in a French processing plant.	Meat processing plant	Transmission and risk factors
Moore et al. (2021) [18]	Yes	UK	Sector-specific study. Responds to the TUC’s calls for a strengthened health and safety agenda, improved safety guidance and tougher regulatory actions in the light of COVID-19.	Food and drinks sector	Transmission, prevention and risk
Mutambudzi et al. (2020) [19]	Yes	UK	Multiple sector study—investigates severe COVID-19 risk by occupational group.	Process, plant and machine operatives	Transmission
Nakat and Bou-Mitri (2021) [29]	Yes	Global	Literature review—aims at assembling all current knowledge about COVID-19 and its impact on the food industry.	Food sector	Prevention
Nafilyan et al. (2021) [20]	Yes	England, UK	Multiple sector study—analyses occupational and COVID-19 mortality in England.	Food production	Mortality
Office for National Statistics (2021) [21]	Yes	UK	Epidemiological surveillance data—reports on COVID-19 related mortality rates within different occupations between March and December 2020.	Various	Transmission/cases/mortality
Rizou et al. (2020) [30]	Yes	Global	Literature review—summarises possible transmission routes of COVID-19 through the food supply chain.	Food sector as whole	Transmission and prevention
Rubenstein et al. (2020) [12]	Yes	Maryland, USA	Sector-specific study. Investigates the factors contributing to the transmission of COVID-19 within foreign-born workers.	Catering and food sector	Transmission and prevention
Steinberg et al. (2020) [13]	Yes	South Dakota, USA	Sector-specific study. Investigates COVID-19 outbreak among employees at a meat processing facility.	Meat processing plant	Transmission
The national COVID-19 outbreak monitoring group (2020) [24]	Yes	Spain	Epidemiological surveillance data—reports on outbreaks notified to the national level in Spain during the summer of 2020.	Meat processing plant	Transmission
Vanderwaal et al. (2021) [14]	Yes	USA	Sector-specific study—Examined PCR testing and modelled transmission at pork plants in the US.	Pork processing plants	Transmission and prevention
Walshe et al. (2021) [23]	Yes	Ireland	Sector-specific study. Provides retrospective outbreak investigation in a meat processing plant and a description of the measures taken to prevent or contain further outbreaks	Meat processing plant	Transmission and Prevention
Waltenburg et al. (2021) [15]	Yes	USA	Sector-specific study. Describes COVID-19 among US food manufacturing and agriculture workers.	Food processing, manufacturing and agriculture workplaces	Transmission and prevention
Zuber & Brussow (2020) [31]	Yes	Global	Literature review addressing the presence and persistence of COVID-19 in the food environment.	Food sector as a whole	Prevention

**Table 2:** Summary of study findings for COVID-19 related cases and infection and mortality rates.

Study	Sector Facility	COVID-19 Related Cases or Outbreaks	COVID-19–Related Deaths	Time Period	Other
Billingsley et al. [27]	Food packing	n/a	0	12 March 2020–23 February 2021	n/a
Bui et al. [7]	Manufacturing sector and wholesale trade	Manufacturing—467 (34%) Wholesale trade—200 (14%)	Manufacturing—12 (3%) Wholesale trade—3 (2%)	6 March 2020–5 June 2020	n/a
Chen et al. (a) [8]	Food and Agriculture	n/a	1050 (897–1204) (excess deaths)	March 2020–October 2020	n/a
Chen et al. (b) [16]	Manufacturers and packers of Food	117/1317 outbreaks (9%) 6998 total workplaces in this category	n/a	18 May 2020–12 October 2020	Outbreak rate: 1672/100,000
Dyal et al. [9]	Meat and Poultry processing	4913 (3.0%) (Total across 19 states)	20 (0.4%) (Total across 19 states)	April 2020	n/a
Herstein et al. [10]	Meat processing	5002 of 26,000 (0.192%)	n/a	April 2021	n/a
Hiironen et al. [17]	Food production and agriculture	—	n/a	late August, late September, and late October 2020	Odds ratio 1.03 (95% CI 0.60 to 1.78) comparing infection in food production and agriculture compared to other workers.
House et al. [11]	Meat packing	Out of 582 patients in the ED, 74% of meat packing plant exposed patients tested positive for COVID-19, while 12% of those without a meat packing plant exposure tested positive.	n/a	March 2020–May 2020	n/a
Kotsiou et al. [26]	Food production sector	(pre lockdown) 17 of 48 (35%) (during lockdown) 1 out of 17 (5%)	n/a	2 sets–one before lockdown (5–6 November 2020) and one month after the lockdown initiation (30 November–1 December 2020)	n/a
Mallet et al. [22]	Meat processing plant	140 cases among 1347 workers, 87.5% of which were tested	0	May 2020	n/a
Mutambudzi et al. [19]	Process, plant and machine operatives	17 out of 4775 (0.4%) with “severe” COVID-19	n/a	16 March 2020–26 July 2020	Relative risk 1.12 (95% CI 0.52 to 2.42) comparing risk of severe COVID-19 for food workers compared to non-essential workers.
Nafilyan et al. [20]	Food sector	n/a	n/a	24 January 2020–28 December 2020	Hazard ratio 1.15 [95% CI 0.89 to 1.50] (men) 1.15 [0.750–1.77] (women) These compare mortality in food sector to non-essential workers and are adjusted for multiple demographic factors.
Office for National Statistics [21]	Process, plant and machine operatives	n/a	827 deaths for men (52.8 deaths per 100,000 males) 57 deaths for women (33.8 per 100,000 )	March 2020–December 2020	n/a
Steinberg et al. [13]	Meat processing plant	929 cases among 3635 workers (25.95%)	n/a	March 2020–April 2020	n/a
The national COVID-19 outbreak monitoring group [24]	Slaughterhouses/meat plants and Fruit and Vegetable sector	Slaughterhouses/meat plants—767 cases Fruit and Vegetable sector—500 cases (total number of workers not available)	n/a	May 2020	n/a
Walshe et al. [23]	Meat processing plant	107 cases among 290 workers	n/a	Mid to late 2020	n/a
Waltenburg et al. [15]	Food manufacturing and agriculture workplaces	8978 cases among workers in 742 food manufacturing and agriculture workplaces in 30 states Among 15 states that reported worker populations in affected workplaces, 8.2% of 30,609 workers received COVID-19 diagnoses	55 (0.6%)	1 March 2020–31 May 2020	n/a
VanderWaal et al. [14]	Pork-processing plants	Cumulative incidence of clinical (PCR-confirmed) disease plateaued at ~2.5% to 25% across the three plants studied.		March 2020–August 2020	n/a

**Table 3:** Summary of findings on risk factors.

Study	Risk Factor Identified	Findings
Aday & Aday [28]	Transport Income/sick pay Environmental factors	Employees within food factories are more likely to share the same buses or use car-sharing systems, which they state allowed the virus to spread further within the community. majority of workers in the food manufacturing sector have lower income and do not have health insurance/paid sick leave cold and dark environments without any ultraviolet light can keep the virus alive for several hours, resulting in further transmission (not food sector specific).
Anand et al. [32]	Transport	Analysed survey results from 2000 respondents in the USA and UK. Found that workers who were more likely to use public transport or share cars were at higher risk of catching COVID-19.
Bui et al. [7]	Ethnicity	Only 24% of workers in Utah’s 15 affected sectors identified as Hispanic or Latino, or another race apart from white, however, 73% of all the workplace outbreak-associated COVID-19 cases were within these ethnic groups. The racial and ethnic disparities in workplace outbreak-associated COVID-19 cases found in Utah and identified in meat processing facility outbreaks in other states demonstrate a disproportionate risk for COVID-19. These disparities might be driven, in part, by longstanding health and social inequities, resulting in the overrepresentation of Hispanic and non-white workers in frontline occupations. Hispanic and non-white workers have less flexible work schedules and fewer telework options compared with white and non-Hispanic workers.
Chen et al. (a) [8]	Ethnicity	The pandemics effect on mortality was highest for Latino and black workers in this sector, who had a 59% increase in mortality when compared to other ethnic groups. Variation by race/ethnicity may also reflect variability of risk within an occupation. For example, one job title may have higher risk within one sector than in another, or one manufacturing environment may be better ventilated or have better access to personal protective equipment than another.
Rubenstein et al. [12]	Ethnicity Transport	The odds of foreign-born workers commuting to work with individuals from outside their household was around 1.9 times the odds for US-born workers. Foreign-born workers were more likely to be disproportionately placed in certain areas and jobs. E.g., they were more likely to work in cold-temperature areas. Among the 359 out of 2345 workers interviewed, 35.7% commuted to work via shared transport with persons from outside their household. Structural factors were more apparent than were behavioral factors, especially among foreign-born workers. Some structural factors (e.g., shared transportation and larger household size) are common features of foreign-born populations in the United States.
Kotsiou et al. [26]	Ethnicity	High number of foreign-born workers working in food production sector in Greece (a sector which had some of the highest number of positive COVID-19 results)
Mallet et al. [22]	Ethnicity Transport Shared-accommodation Temporary-workers	Foreign-born workers accounted for half of the total COVID-19 cases, and 95.2% of these workers worked in the deboning and cutting department. 62 cases (52.5%) reported carpooling or sharing their accommodation. These were both more frequently reported by Eastern European cases. The investigation of the outbreak revealed a significantly increased risk of SARS-CoV-2 infection for workers of subcontractors and some foreign-born workers.
Mutambudzi et al. [19]	Ethnicity	Non-white essential workers had the highest risk of COVID-19 (risk ratio of 8.34) when compared to white essential workers, including within the food and plant and machine operatives.
Moore et al. [18]	Income/sick pay	Of the workers who were required to self-isolate, one in five did not receive sick pay 25% of their worker survey respondents in food manufacturing factories reported changes to sick pay, while 25% reported changes to sickness absence These changes included over one in four managers reporting that there had been an increase of 34% in sick pay for food manufacturers.
Günther et al. [25]	Environmental factors	Found that environmental conditions, including low temperature, low air exchange rates, air recirculation, along with lack of social distancing between workers, created an “unfavourable mix of factors promoting efficient aerosol transmission SARS-CoV-2 particles” Transmission of the virus can occur over distances of at least 8 metres in confined spaces, particularly in conditions with low air exchange and high rates of recirculated unfiltered air. Study implicates that common operational conditions in industrial meat processing plants promote the risk of SARS-CoV-2 super spreading events.
Herstein et al. [10]	Ethnicity	Higher risks of poor outcomes among ethnic and racial minority groups in meat-processing facilities across the state of Nebraska, with evidence showing that 67% of confirmed cases in this sector were individuals who were Hispanic or Latino. Ethnic and racial minorities also constituted 73% of hospitalised cases, 78% of ICU admissions and 86% of deaths
House et al. [11]	Ethnicity	Patients from meatpacking plants were more likely to be Black or Hispanic than the emergency department patients without the occupational exposure Although only 8.2% of people in the emergency department stated that their exposure was potentially from working in a meat packing facility, 60% of these individuals were of Hispanic ethnicity, compared to 10% of patients without this exposure.
Steinberg et al. [13]	Environmental factors Contract workers	Highest risk areas of the meat processing facility were the Cut, Conversion and Harvest department-groups, all of which had numerous employees who were working with less than 2 m distance between them. Cases were higher amongst nonsalaried individuals.
Walshe et al. [23]	Environmental factors	After carrying out air quality monitoring in the boning hall and abattoir of a meat processing plant, it was found that the boning hall had showed a gradual build-up of carbon dioxide and aerosol particles over the course of a work shift. They confirmed that this area was poorly ventilated and was highly favourable for aerosol transmission of COVID-19. On the contrary, CO_2_ concentration in the abattoir showed a marked decrease during the working shift and increased during the working day. However, the number of fluorescent particles was low and showed no significant change over time. The average air temperatures were 10 °C in the boning hall and 18 °C in the abattoir. The relative humidity was higher on average in the abattoir (71%) than in the boning hall (66%).
Waltenburg et al. [15]	Ethnicity	Higher number of confirmed COVID-19 cases amongst Hispanic and Latino workers, (72.8% of overall cases) within the food manufacturing and agriculture workplaces. 83.2% of cases occurred among racial and ethnic minority workers Racial and ethnic distribution of meat and poultry processing workers with COVID-19 differed slightly, with a higher percentage of cases being reported among non-Hispanic Black and non-Hispanic Asian/Pacific Islander workers. Study supports findings from prior reports that part of the disproportionate burden of COVID-19 among some racial and ethnic minority groups is likely related to occupational risk

**Table 4:** Summary of main risk mitigations found in the literature.

Risk Mitigation	Findings
Testing/screening	Rapid antigen testing is crucial in providing infection control within different occupations and should be offered to all workers regularly. However, this can also produce false negatives/false-positive tests and fear/stigma of positive COVID-19 cases [26]. An increase in the uptake of visitor screenings at food production sites is essential for visitors, service providers, suppliers, delivery drivers, pest control, etc. [28,29]. While transmission slowed amongst all the pork processing plants when routine PCR testing was put into place, it was mainly due to other biosafety measures employed at different plants and the possibility of herd immunity within the workforces [14].
Ventilation	Increasing the number of air exchanges per hour and installing high efficiency particulate air (HEPA) filtration should be considered as one of the “most effective engineering control for COVID-19 (although) more study is needed on aerosol transmission dynamics in this setting” [10]. EU food hygiene legislation requires that meat cutting rooms are maintained at a temperature of <12 °C. However, it is important to research if meat cutting could be performed in rooms operated at a higher ambient temperature without compromising on food safety. Where possible, carbon dioxide concentrations should also routinely be used [23]. Ventilation should be maximised within indoor work settings, as SARS-CoV-2 transmission can occur in a crowded and poorly ventilated space where viral concentrations within the room may raise to levels similar to that of exhaled air by COVID-19 patients [31]. However, this was not analysed in a food production setting.
Sick pay	Offering sick pay and flexible working schedules for workers is essential and can help reduce the racial disparities between ethnic minority workers and white workers that can currently be seen in the number of COVID-19 cases within the food sector [7].
Social distancing	Incidence of COVID-19 cases reduced in 62% of studied meat processing facilities after the adoption of universal masking and physical barrier interventions. However, while physical barriers may help limit spread, the low temperatures and limited fresh air supply in meat processing factories could facilitate longer-range aerosol transmission, hence increasing risk of infection amongst workers [10]. Separating employees with a minimum of 1–2 metre space were found by Nakat and Bou-Mitri [29] and Zuber and Brussow [31] as effective ways of limiting the spread of COVID-19. Facilities should consider reducing work hours, rotating shifts and placing workers into bubbles so that more social distancing and better tracking of cases can take place [28].
Adequate hygiene practices	Nakat and Bou Mitri [29] and Rizou et al. [30] recommended laundry cleaning clothes after work shifts, identifying and disinfecting high-touch surfaces in the food facilities. Studies they reviewed also recommended minimising tool sharing and disinfecting equipment multiple times in a shift for items that must be shared/used by more than one person. Frequent hand washing is essential [7].
PPE	The implementation of face masks in meat-processing facilities would only work if further education was also provided to employees on the topic [10]. Use of face masks should be considered as a complementary measure and not as a replacement for established preventative measures [31]. 25% of workers reported that their employer had not provided sufficient PPE in March/April 2020, while some managers also stated that they did not believe sufficient PPE was available in their workplace during this time.
Other	Educational risk mitigation strategies, in the form of posters (in several languages), explanation of COVID-19 symptoms, information about isolating and ensuring risk mitigation is also controlled in the community can all help significantly reduce COVID-19 outbreaks and cases in Meat processing plants [23].

## Data Availability

All data underlying the results are available as part of the article and no additional source data are required.
